# The Long and Winding Road

**DOI:** 10.5812/traumamon.7536

**Published:** 2012-10-10

**Authors:** Mohammad Hosein Kalantar Motamedi

**Affiliations:** 1 Trauma Research Center, Baqiyatallah University of Medical Sciences, Tehran, IR Iran

**Keywords:** Students, Education, Medicine

Day by day the competition get tougher, the exams harder and the textbooks larger. Indeed, it is a long and winding road for the high school graduate seeking university admission to study medicine. The graduate desiring to excel must prepare to travel a tortuous path laden with obstacles. In Iran, in order to obtain admission to medical school, one must place approximately among 3622 of the 442,000 or so participants in the Annual Nation-wide Medical University Entrance Exam (1). If accepted, the 18 year-old high school graduate must then complete 7 years of medical school to get a doctorate degree in medicine (MD). The general practitioner (GP) then 25 years-old, must compete again to get accepted in the Annual National Residency Exam in order to pursue a medical specialty. Pursuit of a specialty is essential because the large cities of the country now have a surplus of GPs; and more patients opt to refer to a specialist for their ills rather than to a GP. Today, even in remote rural areas, more and more patients are referring to specialists in nearby cities instead of local GPs when seeking treatment. This issue compels GPs to seek higher education. If accepted in an accredited residency program, the GP must study another 4-6 years depending on the specialty. After completion of residency, the specialist then aged between 29-31 yearsold often seeks a fellowship or subspecialty; because the major cities of Iran are now also replete with specialists in almost all fields of medicine; and city residents refer to subspecialists. Fellowships takes another 1-3 years to complete. After completion of a fellowship, the subspecialty physician at the age of 30-34 is finally ready to open an office and start a private practice from ground zero, provided of course, the doctor is not male. Because if the doctor is male he must first serve the 18-21 month mandatory military service upon completion of which, he will then be between 32-36 years-old. After all this hard work, dedication, mental and physical devastation and all the blood, sweat and tears, those who make it to the top look back in time, and deliberate on common questions was it all really worth it ? Would another profession have been better ? Should our children pursue this career and can they take all the mental and physical stress, sleepless nights and high workload inherent to the medical profession? Those who do not make it through college often drop out in medical school and many that just can not hack the competition practice as GPs or drive taxis in Tehran to make a living. So what is the bottom line ? Well, I think high school graduates should be well briefed with these issues via guidance counselors before they decide to hit the road (Figure 1)(2-4).

**Figure 1:**
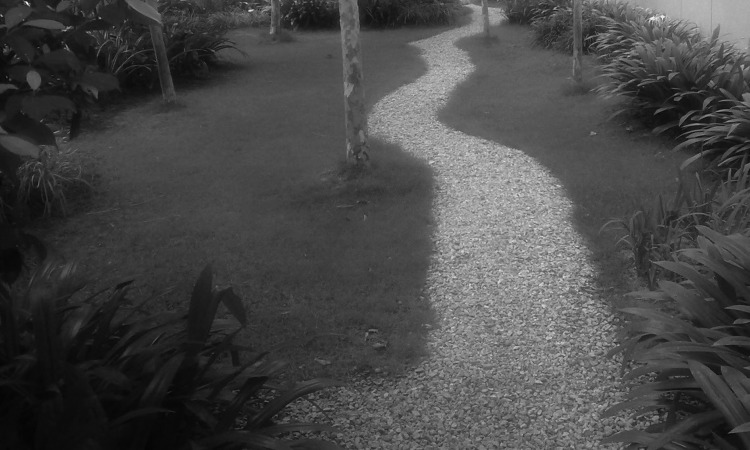
The Long and Winding Road

## References

[1] (2012). Evaluation Organization of Iran. Tehran. http://www.sanjesh.org.

[2] (2012). Wikipedia, the free encyclopedia. Medical education. http://en.wikipedia.org/wiki/Medical_education.

[3] Azizi F (1997). The reform of medical education in Iran.. Med Educ.

[4] Gharib R (1966). A report on medical education in Iran.. J Med Educ.

